# GPX4 predicts poor prognosis and regulates tumor proliferation and senescence in colorectal adenocarcinoma

**DOI:** 10.32604/or.2025.063395

**Published:** 2025-07-18

**Authors:** YU ZHANG, QINGKUN WANG, YUE HAN, JUNJIE PIAO, XIUYING JIN

**Affiliations:** 1 Department of Oncology, Affiliated Hospital of Yanbian University, Yanji, 133002, China; 2 Key Laboratory of Pathobiology (Yanbian University), State Ethnic Affairs Commission, Yanji, 133002, China

**Keywords:** Glutathione peroxidase 4 (GPX4), Colorectal adenocarcinoma (COAD), Prognosis, Proliferation, Cellular senescence

## Abstract

**Background:**

Colorectal adenocarcinoma (COAD) is one of the most common gastrointestinal malignancies. There is a pressing need to recognize reliable biomarkers that can improve diagnostic accuracy, predict prognosis, and serve as effective molecular targets. Glutathione peroxidase 4 (GPX4) is an important antioxidant protein. Evidence demonstrates that abnormal expression of GPX4 is related to cancer initiation and progression. However, the role of GPX4 in COAD remains unclear.

**Methods:**

We employed bioinformatics analysis and conducted subsequent validation of biological processes, including cell counting kit-8 assay (CCK-8), colony formation assay, reverse transcription-quantitative polymerase chain reaction (RT-qPCR), 5-ethynyl-2′-deoxyuridine assay (EdU), western blot, immunohistochemistry, senescence associated β-galactosidase (SA-β-gal) staining and immunofluorescence to explore the expression status, prognostic value and biological function of GPX4 in COAD.

**Results:**

Our data revealed that GPX4 mRNA expression was upregulated in COAD tissues and could predict the prognosis in patients with COAD. High GPX4 expression was associated with increased infiltration of malignant cells. We also performed a series of cell experiments confirming that GPX4 knockdown inhibited proliferation and induced cellular senescence, as determined by using CCK-8, colony formation, and EdU assay. In addition, SA-β-gal staining and senescence-associated secretory phenotype (SASP) components, such as P21 and Interleukin-6 (IL-6), were increased in GPX4 knockdown cells, while Lamin B1 was decreased. Moreover, we predicted that high expression of GPX4 was related to low immune cell infiltration.

**Conclusion:**

This study demonstrates that GPX4 is a potential prognostic biomarker and target gene for COAD.

## Introduction

Colorectal adenocarcinoma (COAD) is one of the most common gastrointestinal malignancies. In 2020, across the world, the number of Colorectal cancer (CRC) cases was quite high. Specifically, there were 1.9 million new cases and 0.935 million deaths. These amounts respectively accounted for 10.0% and 9.4% of the aggregate cancer cases [1]. The 5-year survival rate for CRC exceeds 50%; however, for metastatic CRC, it drops dramatically to just 12%, due to the presence of unresectable metastases [2,3]. Nearly one-quarter of CRC cases are diagnosed at an advanced stage [4–6]. Hence, there is a pressing need to recognize reliable biomarkers and effective molecular targets.

Glutathione peroxidase 4 (GPX4) is an important antioxidant protein in mammalian cells. The unique function of GPX4 is the reduction of lipid hydroperoxide products on the cell membrane to nontoxic lipid alcohols, using glutathione as a cofactor [7–9]. Abnormal expression of GPX4 is associated with various human diseases, such as alcoholic liver disease [10], and age-related cataracts [11]. Recent studies have also demonstrated that abnormal expression of GPX4 was related to cancer initiation and progression. For instance, Sha et al. reported that higher GPX4 expression strongly correlated with better distant metastasis-free survival in patients with breast cancer [12]. However, Chen and colleagues presented opposite results, finding that increased GPX4 expression promotes thyroid cancer tumorigenesis [13]. Meanwhile, Ren et al. also found that higher protein expression of GPX4 was related to poor prognosis in gliomas [14], suggesting that GPX4 plays different roles in different cancers.

Present studies have also shown that an increase in GPX4 is also associated with the protection of cancer cells from cellular senescence and immune evasion. Such as the SLC7A11/GPX4 axis has been reported to play a role in cellular senescence [15]. In addition, Chen et al., who found that GPX4 is related to the activation of the cGAS-STING pathway, suggest that GPX4 is also associated with immune evasion of cancer [16]. Moreover, numerous treatments, including small-molecule inhibitors, natural products, and gene deletions, have been identified to suppress cancer progression by targeting GPX4 in CRC [17]. These results suggest that GPX4 plays a crucial role in cancer initiation and progression.

In this study, we investigated the role of GPX4 in the progression of COAD. We measured the expression levels of GPX4 in COAD tissues and evaluated the relationship between GPX4 expression and patient prognosis. In addition, we assessed the effect of GPX4 on proliferation and cellular senescence in COAD. Furthermore, we predicted the relationship between GPX4 expression and immune cell infiltration. Taken together, our results provide novel insights into how GPX4 promotes cancer progression in COAD and suggest its potential as a biomarker for predicting prognosis.

## Materials and Methods

### Human tissues

All samples were obtained from nine primary colorectal cancer specimens, comprising five cases of right hemicolectomy and four cases of left hemicolectomy. These samples were surgically resected by surgery at Affiliated Hospital of Yanbian University with no preoperative chemotherapy, radiotherapy, or other tumor-specific treatments, and the study was approved by the Ethics Committee of Affiliated Hospital of Yanbian University (No. 2024673) (Yanji, China). Each sample consisted of one specimen of colorectal cancer and one specimen of normal adjacent tissue. Each sample was collected from 5 cm away from the lesion. Specimens were collected under aseptic conditions in the operating room within 5 min of surgical resection. Each sample was confirmed by a pathologist after the operation. Written informed consent was obtained from all the study subjects before enrollment.

### Cell culture and transfection

Human CRC cell lines SW620 and SW480 (C5227; C5233, Zhejiang, China) were purchased from Baidi Biotech Ltd., Hangzhou, China. The cell lines were cultured in DMEM (Absin, abs9560, Shanghai, China) with 1% penicillin/streptomycin (Solarbio, P7630, Beijing, China) and 10% fetal bovine serum (BIOFIL, FBS111025, Guangzhou, China). The cell lines were grown in a humidified environment with 5% CO_2_ at 37°C. Mycoplasma contamination was routinely tested by PCR analysis. Small interfering RNAs (Si-Con, Si-GPX4#1, Si-GPX4#2, and Si-GPX4#3) targeting GPX4 were designed and synthesized by IBSBIO (Shanghai, China). SW620 and SW480 cells were transfected in 6-well plates via Lipofectamine™ 3000 (Invitrogen, L3000015, Carlsbad, CA, USA). After transfecting for 24 h, use for further experiments. The detailed sequence information is summarized in Table 1.

**Table 1:** Sequences of the siRNAs used in the experiments

Primer	Nucleotide Sequence (5′−3′)
Si-Con (NC)	Sense: 5′-UUCUCCGAACGUGUCACGUTT-3
	Anti-sense5′-ACGUGACACGUUCGGAGAATT-3
Si-GPX4#1	Sense: 5′-CAGGGAGUAACGAAGAGAUUU-3
	Anti-sense: 5′-AUCUCUUCGUUACUCCCUGUU-3′
Si-GPX4#2	Sense: 5′-GAGGCAAGACCGAAGUAAACU-3
	Anti-sense: 5′-UUUACUUCGGUCUUGCCUCAC-3′
Si-GPX4#3	Sense: 5′-UUCGAUAUGUUCAGCAÀGAUU-3
	Anti-sense: 5′-UCUUGCUGAACAUAUCGAAUU-3′

### CCK-8 assay

Briefly, CRC cells transfected with si-GPX4 were seeded in 96-well plates (5 × 10^3^ cells/well). The next day, 100 μL of medium containing 10 μL of CCK-8 reagent (Invigentech, 1V08-500, Irvine, CA, USA) was added to the cell culture medium and further incubated for 2 h in the 37°C incubator. The relative absorbance was measured at 450 nm to calculate the viability of cells.

### EdU assay

Briefly, CRC cells transfected with si-GPX4 were seeded in 96-well plates (2 × 10^4^ cells/well). The proliferation capacity of COAD cells was assessed using the EdU Kit according to the manufacturer’s instructions (RiboBio, C10310-1, Guangzhou, China). Images were captured using a fluorescence microscope (Olympus, IX73, Tokyo, Japan) and further analyzed using ImageJ software (version 1.52, National Institutes of Health, Bethesda, MD, USA).

### Colony formation assay

Briefly, CRC cells transfected with si-GPX4 were plated in 6-well plates (800 cells/well) and incubated for 13 days. The cells were then washed with phosphate-buffered saline (PBS) (Solarbio, P1022). The formed transfected cell colonies were fixed with 4% paraformaldehyde (Beyotime, P0099, Huaian, China) and stained with 0.1% crystal violet (Solarbio, G1063). The acquired images were analyzed using ImageJ software.

### Determination of SA-β-gal activity

To observe positively senescent cells, the SA-β-gal Staining Kit (Beyotime, #C0602) was used. Cells were grown to 30%–50% confluence, washed with 2 mL of PBS, and the fixative was applied for 15 min. After that, the staining working liquid was used to incubate the cells at 37°C overnight. The next day, cell morphology was observed using a light microscope (Olympus, IX73). The number of stained positive cells in the field of view was counted, with the blank group used as a control.

### Total protein extraction and western blot

Whole transfected cells were collected after rinsing with PBS three times. The cell lysates were collected on ice using radio-immunoprecipitation assay lysis buffer (RIPA) (Solarbio, R0020) containing protease and phosphatase inhibitors (Solarbio, P1260), and the extract was collected by centrifugation at 12,000 rpm (HITACHI, CT15RE, Tokyo, Japan) for 30 min at 4°C. When extracting proteins from human tissue samples, we placed the tissues in the RIPA containing protease and phosphatase inhibitors and ground them using a tissue homogenizer. The supernatant was collected as the total lysate protein. After quantitation and denaturation, the samples (15 g protein/well) were separated by 10% SDS-PAGE gel electrophoresis (Epizyme, PG112, Shanghai, China) and transferred to a Polyvinylidene fluoride (PVDF) membrane (Merck, IPVH00010, Darmstadt, Germany), followed by incubation with primary antibodies and gentle shaking at 4°C overnight. The next day, secondary antibodies (1:3000 ProteinTech, SA00001-1/SA00001-2, Chicago, IL, USA) were incubated for at least 70 min with gentle shaking at room temperature (RT). All protein bands were detected using a chemiluminescence reagent (ZOMANBIO, ZD310-2, Beijing, China) after they were rinsed three times with Tris-buffered saline having Tween-20 (Beyotime, ST1727). The following antibodies were used: anti-GPX4 (1:1000 ProteinTech, 30388-1-AP), anti-glyceraldehyde-3-phosphate dehydrogenase (GAPDH; 1:3000 ProteinTech, 10494-1-AP), anti-HSP90 (1:2000 ProteinTech, 13171-1-AP), anti-interleukin (IL)-6 (1:2000 ProteinTech, 21865-1-AP), anti-β-actin (1:2000 ProteinTech, 66009-1-Ig), and anti-P21 (1:1000 ProteinTech, 28248-1-AP).

### RNA extraction and RT-qPCR

Using commercially available kits, total RNA was extracted from COAD samples and then synthesized (Beyotime, R0027/D7190M), according to the kit instructions. Subsequently, for each cDNA sample, 1 µL of cDNA template, 2 µL of each primer, 7 µL of RNase-free water (ZOMANBIO, ZS105), and 10 µL of SYBR Green PCR mix (Beyotime, D7262) were combined. The total reaction volume for all samples was 20 µL, which was used to calculate the expression of the GPX4 gene through the cycle threshold reading of the corresponding GAPDH. Relative gene expression was evaluated by means of the 2^−ΔΔCT^ method, and all reactions were executed in triplicate. The primers used in this assay were as follows: Human GAPDH (Forward: 5′-GGAGCGAGATCCCTCCAAAAT, Reverse: 5′-GGCTGTTGTCATACTTCTCATGG); Human GPX4 (Forward: 5′-GAGGCAAGACCGAAGTAAACTAC, Reverse: 5′-CCGAACTGGTTACACGGGAA).

### Immunohistochemistry (IHC)

Tissue microarray samples containing 142 clinical information samples were obtained from Shanghai Outdo Biotech Co., Ltd., Shanghai, China (HColA180Su19). For IHC staining, tissue sections were rehydrated in a graded ethanol series after deparaffinisation in xylene. Using microwave heating, sodium citrate buffer (pH 6.0) (Beyotime, P0081) was used for antigen repair and allowed to cool gradually to RT, followed by an endogenous peroxide blocker solution (ZSGB-BIO, PV-9000, Beijing, China) covering the tissue for 25 min. Subsequently, 5% bovine serum albumin (Solarbio, A8020) was used to block non-specific binding. The slides were then incubated with a GPX4 antibody (ProteinTech, 30388-1-AP) at a 1:200 dilution at 4°C overnight. On the morrow, the tissues were incubated in a reaction enhancer (ZSGB—BIO, PV-9000) for 30 min, followed by 20 min incubation with a secondary antibody (ZSGB—BIO, PV-9000) at RT. 3,3′-diaminobenzidine (ZSGB-BIO, ZLI-9018) working solution was added dropwise, and color development was observed under a microscope (Olympus, CX73) for 2–4 min. All tissue sections were counterstained with hematoxylin solution (Solarbio, G1080) for at least 45 s and observed under the microscope (Olympus, CX73) in real time. Finally, neutral gum sealing was performed. The IHC staining results of GPX4 were classified into four semi-quantitative categories according to the proportion of positive cells. Specifically, “−” represented less than 5% positive cells, “+” indicated 6%–25% positive cells, “++” signified 26%–50% positive cells, and “+++” denoted more than 50% positive cells. Positive cell counts >50% were considered strongly positive.

### Immunofluorescence staining

After normal trypsin digestion and centrifugation, 50%–70% of the cells were plated in 6-well plates. The next day, the formed transfected cell colonies which had been formed were fixed using 4% paraformaldehyde for 25 min, and then incubated with 0.5% Triton X-100 (Beyotime, ST1723) for 5 min. Next, the transfected cell colonies were incubated with 5% bovine serum albumin (Solarbio, A8020) for a minimum time of 50 min at RT and incubated with primary antibody (1:300 Lamin B1, ProteinTech, 12987-1-AP) at 4°C overnight. On the morrow, the cells were incubated with the secondary antibody (1:500 ProteinTech, SA00013-4) in the dark for 1 h at RT, counterstained with DAPI (Beyotime, C1025), and imaged using a confocal laser scanning microscope (Olympus, FV3000). ImageJ software was used to analyze the images.

### Bioinformatics analysis

We used the TIMER2.0, SangerBox, XIANTIAO, and GEPIA2.0 databases to comprehensively analyze GPX4 expression and prognosis data [18–20]. GPX4 microarray data (GSE40287, GSE41328, and GSE81582 datasets) were retrieved from the Gene Expression Omnibus (GEO) database. We then used the TISCH (GSE166555 dataset), TISIDB, and TIDE (GSE39582 and GSE12945 datasets) databases to comprehensively analyze the potential interaction between GPX4 and immune infiltration [21–23]. Using the STRING database to construct the protein interaction network [24]. Using the NetworkAnalyst database to construct the transcription factor-miRNA (TF-miRNA) co-regulatory network [25]. Briefly, the results from public databases were obtained using automated statistical analysis.

### Statistical analysis

GraphPad Prism software (version 9.0, GraphPad, USA) and SPSS software (version 26.0, IBM SPSS, USA) were used to analyze the experimental data and tissue microarray samples. The cumulative survival rates of all patients were calculated using the Kaplan-Meier method, and univariate Cox proportional risk regression models were established. The chi-square tests were utilized to assess the correlations between GPX4 expression and clinicopathological characteristics. One-way analysis of variance was deployed to estimate differences among multiple groups. All continuous experimental data were employed in triplicate and expressed as mean ± standard deviation. *p*-value < 0.05 was considered significant.

## Results

### GPX4 mRNA is upregulated in COAD tissues and correlated with poor prognosis

**Figure 1:**
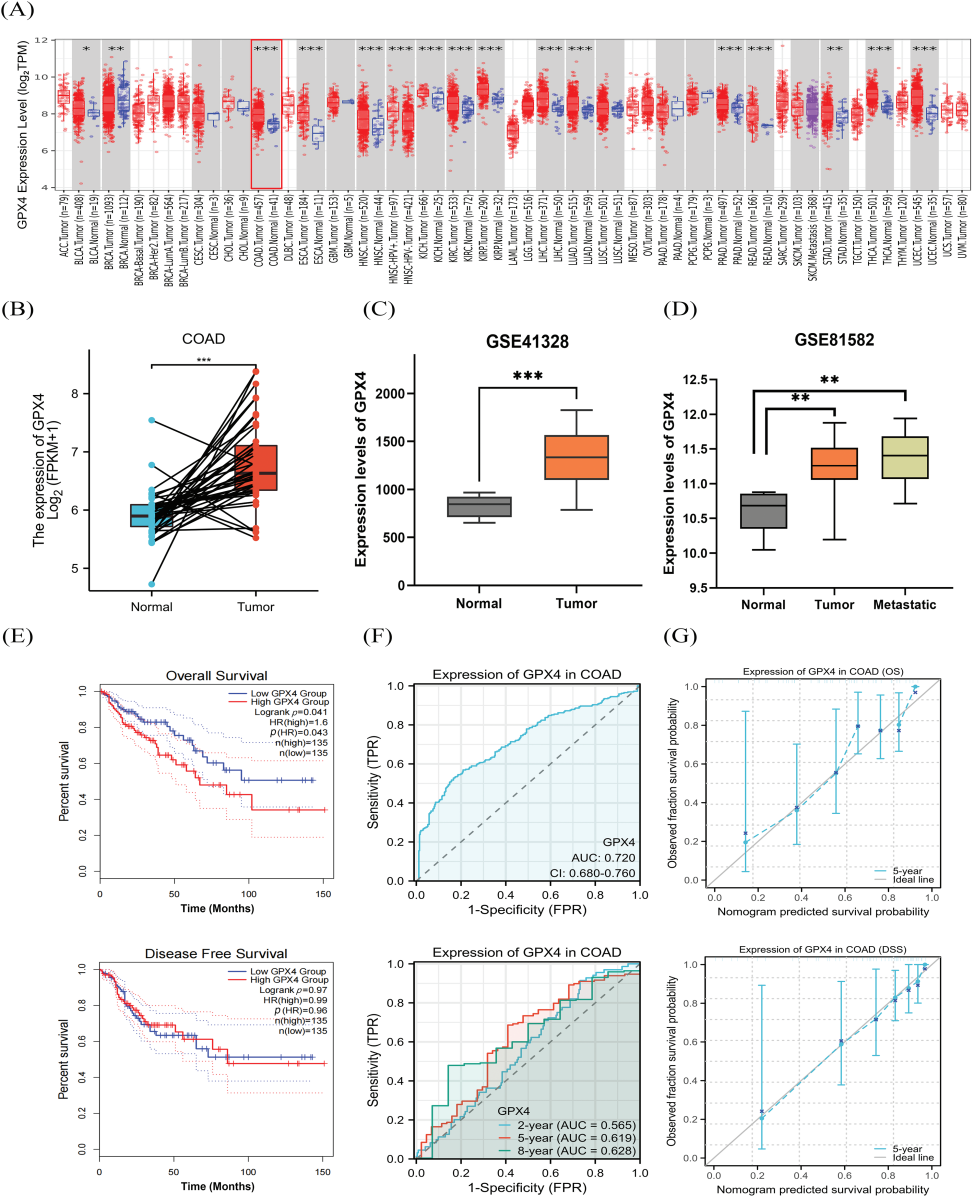
GPX4 mRNA is upregulated in COAD tissues and correlated with poor prognosis. (**A**) The TIMER 2.0 database was utilized to analyze the expression of GPX4 mRNA in pan-cancer and paired adjacent normal tissues. (**B**) Expression of GPX4 in paired normal tissues and cancer samples was analyzed using the XIANTAO platform. (**C**,**D**) GPX4 mRNA expression in normal tissues, primary colorectal adenocarcinoma (COAD) tissues, and metastatic COAD tissues from the GEO databases (GSE41328, GSE81582). (**E**) GEPIA 2.0 was applied to analyze the correlation between GPX4 expression and survival in COAD. (**F**) Receiver operating characteristic curves were employed to determine the diagnostic value of GPX4 in COAD analysis using the XIANTAO platform. (**G**) Diagnostic value of the calibration curves for OS and disease-specific survival of GPX4 in COAD analysis using the XIANTAO platform (*p < 0.05; **p < 0.01; ***p < 0.001).

First, we have detected the expression of GPX4 in pan-cancer tissues using an online database. The TIMER 2.0 database displayed that the expression of GPX4 was substantially upregulated in Bladder Urothelial Carcinoma, Breast invasive carcinoma, COAD, Esophageal Carcinoma, etc. (Fig. 1A). The XIANTAO platform and GEO database were used to predict the expression of GPX4, which confirmed the high expression of GPX4 in COAD (Fig. 1B,C). In addition, GPX4 was expressed at higher levels in primary and metastatic colon cancer tissues than in healthy colon tissues (Fig. 1D). Next, we evaluated the role of GPX4 mRNA expression in predicting pan-cancer prognosis. GPX4 mRNA expression was substantially associated with the prognosis of COAD, Glioma, Liver hepatocellular carcinoma, and other cancers, as determined using the Sangerbox database (Fig. S1A). Consistently, both the GEPIA2.0 and Sangerbox databases predicted that mRNA expression of GPX4 was correlated with short overall survival (OS) in patients with COAD (Fig. 1E; Fig. S1B). Using the XIANTAO database, receiver operating characteristic (ROC) analysis showed that GPX4 had a remarkable level of accuracy in predicting prognosis (AUC = 0.720). Concomitantly, time-dependent ROC analysis also showed that GPX4 had moderate predictive ability for the 2-year (AUC = 0.565), 5-year (AUC = 0.619), and 8-year (AUC = 0.628) (Fig. 1F). The calibration curves showed a high favorable consistency between predicted and observed results (Fig. 1G). These results demonstrate that GPX4 mRNA expression correlates with the prognosis of patients with COAD.

### Construction of the co-regulatory network of GPX4 and GO–KEGG analysis

**Figure 2:**
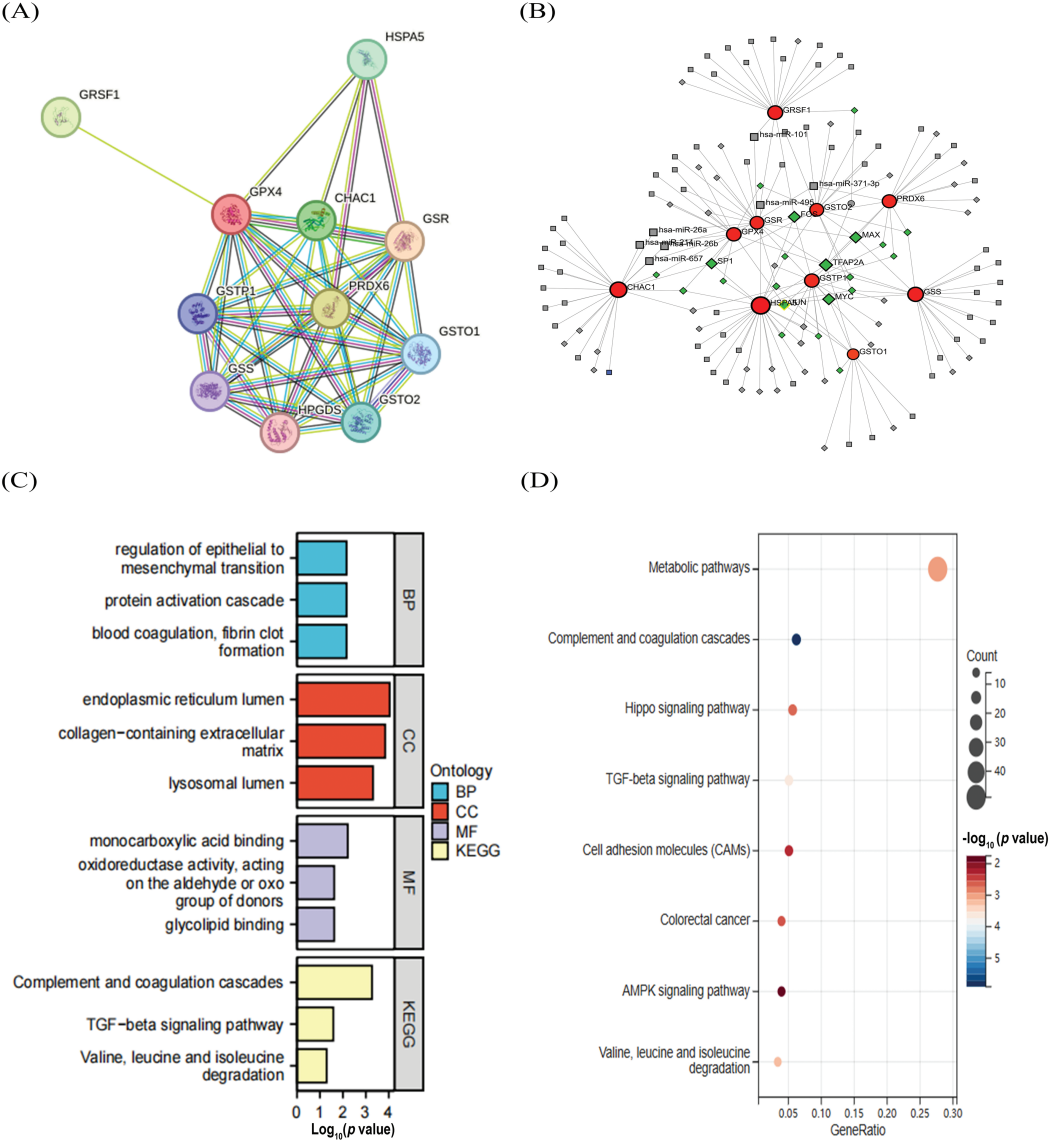
Construction of the co-regulatory network of GPX4 and GO–KEGG analysis. (**A**) GPX4 interaction protein plot. (**B**) TF-miRNA co-regulatory network, comprising 141 nodes and 185 edges (Red: co-expression genes; Gray: miRNAs; Green: transcription factors). (**C**,**D**) GO and KEGG enrichment analyses of inferentially expressed genes obtained from GSE40287 dataset.

To elucidate how GPX4 protein interactions are involved in COAD progression, we used the STRING database to identify 10 hub genes co-expressed with GPX4, including GSR, GRSF1, HPGDS, HSPA5, PRDX6, GSTO2, GSTP1, GSTO1, CHAC1, and GSS (Fig. 2A). In addition, 72 miRNAs that regulated the expression levels of the co-expressed genes were identified by NetworkAnalyst analysis, and the major co-regulated genes were GPX4, HSPA5, GSTO2, GSR, and GSTP1. The miRNAs that hit most commonly were hsa-mir-101, hsa-mir-26a, hsa-mir-26b, hsa-mir-371-3p, hsa-mir-495, and hsa-mir-657. The transcription factors that were hit more commonly were TFAP2A, MYC, FOS, MAX, JUN, and SP1 (Fig. 2B). Moreover, differentially expressed genes (DEGs) were analyzed using the GEO2R tool base on the data obtained from GSE40287 dataset. A total of 364 DEGs were identified in GPX4 knockout group (*p* < 0.05; Fig. S2A). Next, we determined the expression levels of these 10 hub genes and showed that the expression levels of HSPA5, GSTO2, GSTP1, GSTO1, CHAC1, GSS, GSR, and GRSF1 were higher, whereas those of HPGDS and PRDX6 were substantially lower in COAD tissues (Fig. S2B). We then analyzed the association between GPX4 and the above genes in COAD and noted that GPX4 expression had a positive correlation with the expression of GSTO1, GSS, PRDX6, and GSR (Fig. S2C).

GO analysis showed that these DEGs were primarily enriched in monocarboxylic acid binding, endoplasmic reticulum lumen, collagen-containing extracellular matrix, and regulation of epithelial-to-mesenchymal transition (Fig. 2C). KEGG analysis showed that GPX4 may be involved in oncogenesis through regulating the metabolic pathways, complement and coagulation cascades, TGF-β and Hippo signaling pathway (Fig. 2D).

### Knockdown of GPX4 inhibits cell proliferation and induces cellular senescence in COAD

**Figure 3:**
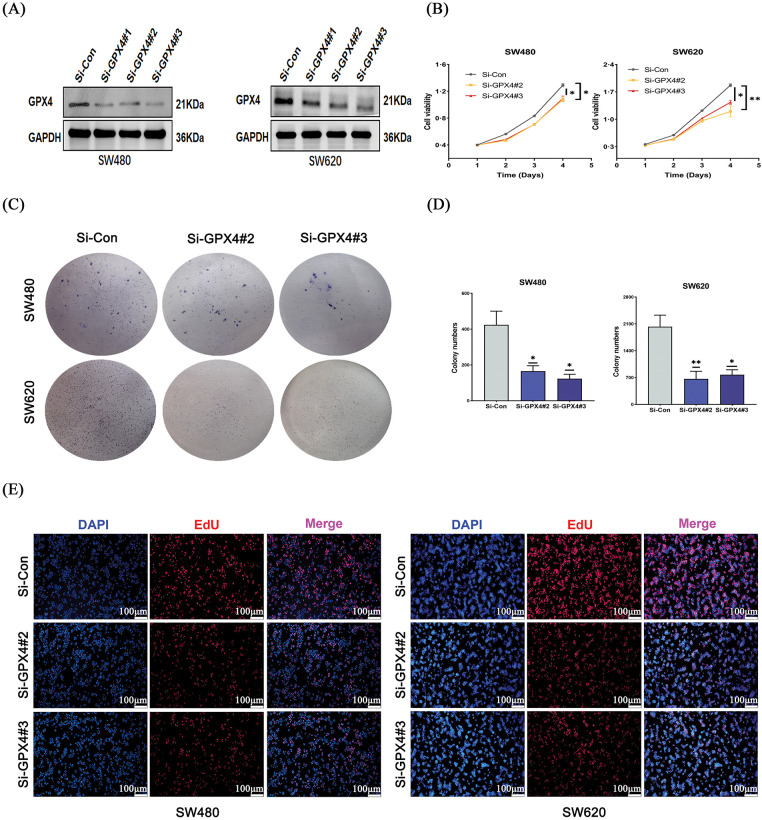
Knockdown of GPX4 inhibits cell proliferation in COAD. (**A**) GPX4 protein expression was determined by western blotting in SW480 and SW620 cells with GPX4 siRNA. (**B**–**D**) CCK8 and colony formation assays were performed to assess the proliferation of SW480 and SW620 cells with GPX4 siRNA. (**E**) Representative images of EdU staining, scale bar = 100 μm (*p < 0.05; **p < 0.01).

To further elucidate the biological function of GPX4 in COAD, SW620 and SW480 cell lines were transfected with si-GPX4 and the effect was confirmed by western blotting (Fig. 3A). CCK-8 assay revealed that cell proliferation was potently inhibited in GPX4 knockdown cells (Fig. 3B). Concurrently, GPX4 knockdown cells showed a marked reduction in colony formation ability (Fig. 3C,D). In addition, GPX4 knockdown inhibited DNA replication in COAD cells, as determined by EdU incorporation assay (Fig. 3E; Fig. S2D). These observations validated GPX4 in the process of regulating COAD cancer cell proliferation.

**Figure 4:**
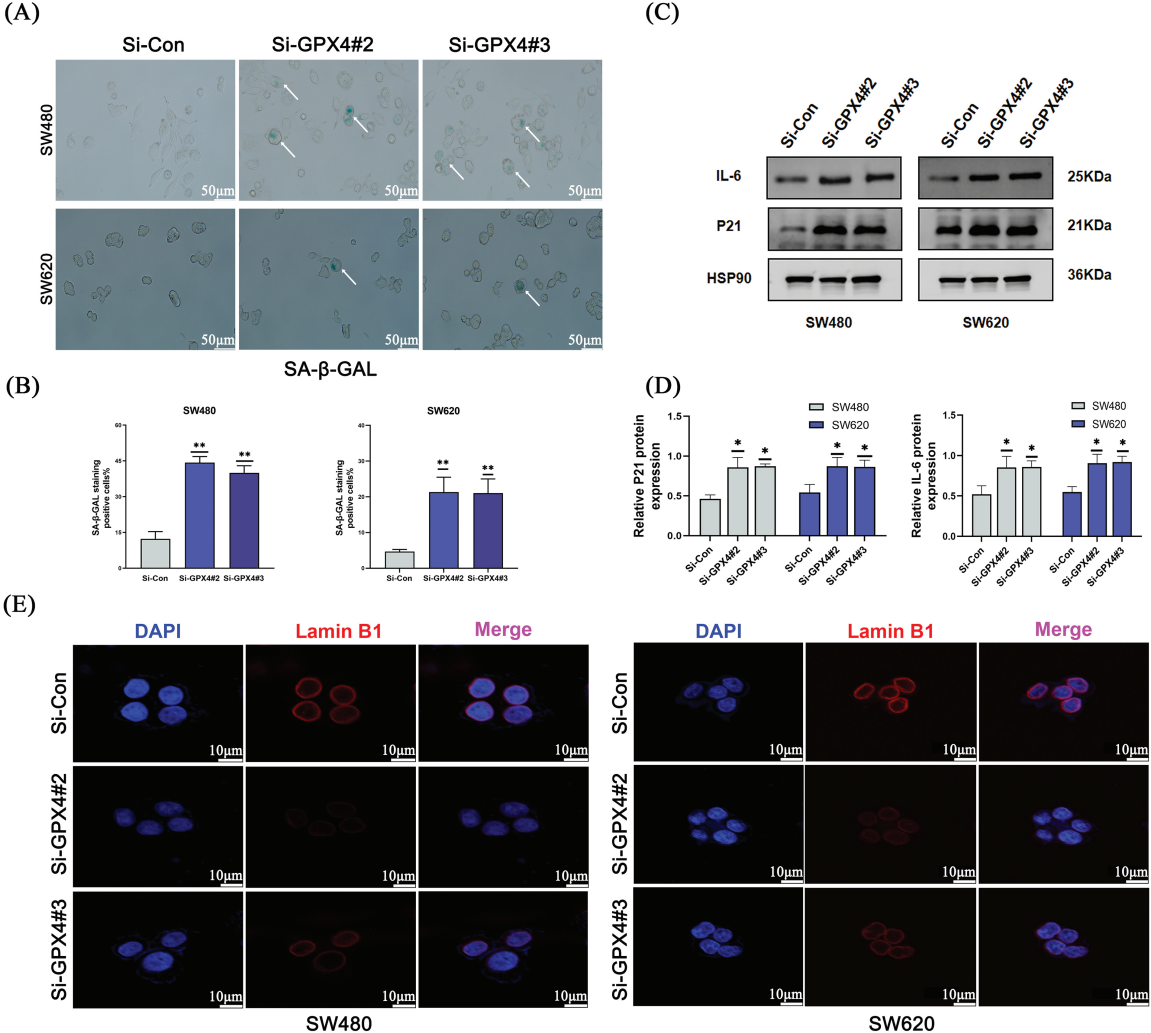
Knockdown of GPX4 induces cellular senescence in COAD. (**A**,**B**) Senescence-associated β-galactosidase staining of SW480 and SW620 cells with GPX4 knockdown, scale bar = 50 μm. (**C**,**D**) Protein expression levels of P21 and interleukin-6 in SW480 and SW620 cells with GPX4 siRNA. (**E**) Lamin B1 immunofluorescence in SW480 and SW620 cells with GPX4 siRNA, scale bar = 10 μm (*p < 0.05; **p < 0.01).

To further understand whether GPX4 could regulate cellular senescence in COAD, SA-β-gal staining was utilized. We observed an increased number of positive regions in the si-GPX4 group (Fig. 4A,B). Consistently, SASP components IL-6, P21, and Lamin B1 was determined after GPX4 knockdown. The expression of Lamin B1 was decreased, while that of IL-6 and P21 was increased in GPX4 knockdown cells (Fig. 4C–E; Fig. S2E). These results demonstrate that GPX4 deficiency leads to inhibited proliferation and enhanced cellular senescence in COAD cells.

### GPX4 protein is overexpressed in COAD and correlates with poor prognosis

**Figure 5:**
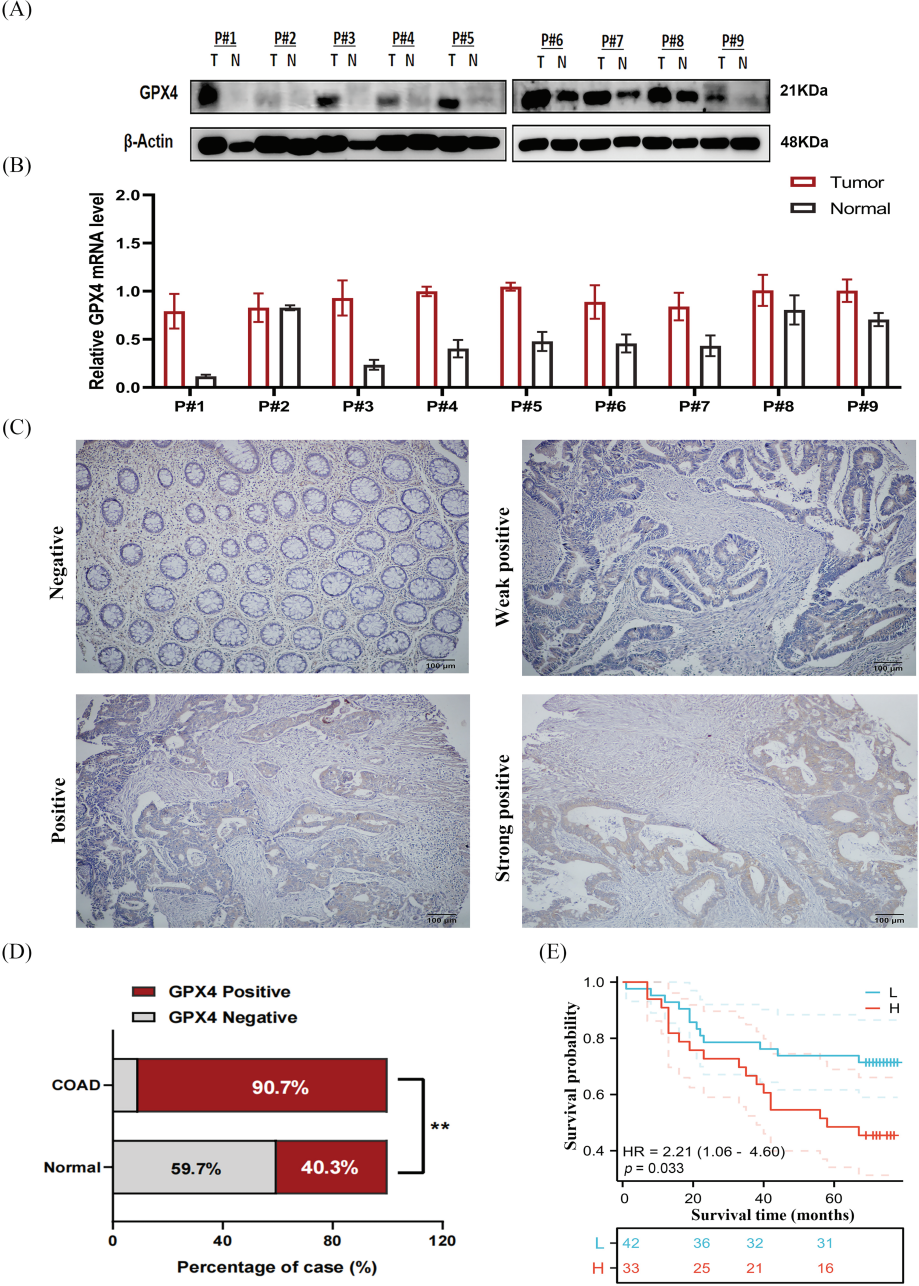
GPX4 protein is overexpressed in COAD and correlates with poor prognosis. (**A**) Protein expression levels of GPX4 in nine paired samples of COAD tissues and adjacent non-tumor tissues. (**B**) mRNA expression levels of GPX4 in nine paired samples of COAD tissues and adjacent non-tumor tissues. (**C**) GPX4 expression in COAD tissues and adjacent non-tumor tissues was determined by immunohistochemistry analysis, scale bar = 100 μm. (**D**) Positive rate of GPX4 expression in COAD and adjacent non-tumor tissues. (**E**) OS of 75 patients with COAD in relation to GPX4 protein expression (**p < 0.01).

To validate the expression status of GPX4 protein in COAD, nine pairs of COAD and adjacent tissues were collected, and the expression of GPX4 was measured using western blotting and RT-qPCR. GPX4 mRNA and protein were overexpressed in eight cases (Fig. 5A,B). Then, IHC staining was performed on tissue microarray, including 75 COAD patients and 67 adjacent non-tumor tissues. As a result, GPX4 was strongly expressed in most COAD tissues but weakly expressed in adjacent tissues (Fig. 5C). The positive rate of GPX4 protein was 90.7% (68/75) (*p* < 0.01), which was obviously higher than that in adjacent tissues (40.3%, 27/67) (Fig. 5D). Kaplan-Meier analysis revealed shorter lifespans in COAD patients with high GPX4 expression than those with low expression (HR = 2.21, *p* < 0.033) (Fig. 5E). Unfortunately, no substantial association was observed between clinicopathological parameters and GPX4 expression in COAD (Table 2). Cox univariate survival analysis indicated that prognosis was influenced by lymph node metastasis (*p* = 0.002), tumor location (*p* = 0.02), clinical stage (*p* = 0.0007), and GPX4 expression. Cox multivariate analysis verified that high levels of GPX4 expression (*p* = 0.033), clinical stage (*p* = 0.036), and tumor location (*p* = 0.036) were strong predictors of prognosis in COAD (Table 3). In brief, these *ex vivo* results demonstrate that the occurrence and development of COAD are inseparable from the high GPX4 expression.

**Table 2:** Correlation between GPX4 expression and clinicopathological parameters of colorectal cancer patients

Clinical features		No. of cases	Strongly positive cases (%)	χ^2^	*p* Value
Age				0.099	0.752
	≤66	39	17 (43.6)		
	66	36	17 (47.2)		
**Tumor size**				0.221	0.637
	≤**3 cm**	8	3 (37.5)		
	**>3 cm**	67	31 (46.3)		
Histological grade				0.215	0.642
	Grade I	15	6 (40.0)		
	Grades II–III	60	28 (46.7)		
**Clinical stage**				0.083	0.773
	T1–3	52	33 (62.3)		
	T4a–4b	23	11 (47.8)		
**LN metastasis**				1.111	0.291
	Absent	51	21 (41.2)		
	Presence	24	13 (54.2)		

Note: *LN metastasis* lymph node metastasis.

**Table 3:** Univariate survival analyses (Cox regression model) of various factors in patients with colorectal cancer.

Factors	B	SE	Wald	HR	95%CI		*p* Value
					Lower	Upper	
**Univariate**							
Age	0.0003	0.0005	0.363	0.9997	0.998	1.000	0.547
Tumor size	0.385	0.399	0.933	0.680	0.311	1.486	0.334
LN metastasis	1.185	0.368	10.344	3.270	1.588	6.370	0.002**
Clinical stage	1.253	0.369	11.544	3.502	1.700	7.218	0.0007**
Histological grade	0.662	0.537	1.520	1.939	0.677	5.560	0.218
Tumor location	0.855	0.374	5.618	2.424	1.166	5.040	0.02*
GPX4	0.872	0.380	5.271	2.391	1.136	5.033	0.022*
**Multivariate**							
Clinical stage	0.871	0.415	4.409	2.390	1.060	5.391	0.036*
GPX4	0.811	0.382	4.515	2.210	1.060	4.601	0.033*
LN metastasis	0.759	0.416	3.335	2.137	0.946	4.827	0.068
Tumor location	0.786	0.375	4.382	2.194	1.051	4.580	0.036*

Note: *p < 0.05, and **p < 0.01. *LN metastasis*: lymph node metastasis. *Tumor location*: sigmoid CRC *vs*. non-sigmoid CRC. *B*: Coefficient. *SE*: standard error. *Wald*: Wald statistic. *HR*: hazard ratio. *CI*: confidence interval.

### The association between GPX4 and immune cell infiltration in COAD

**Figure 6:**
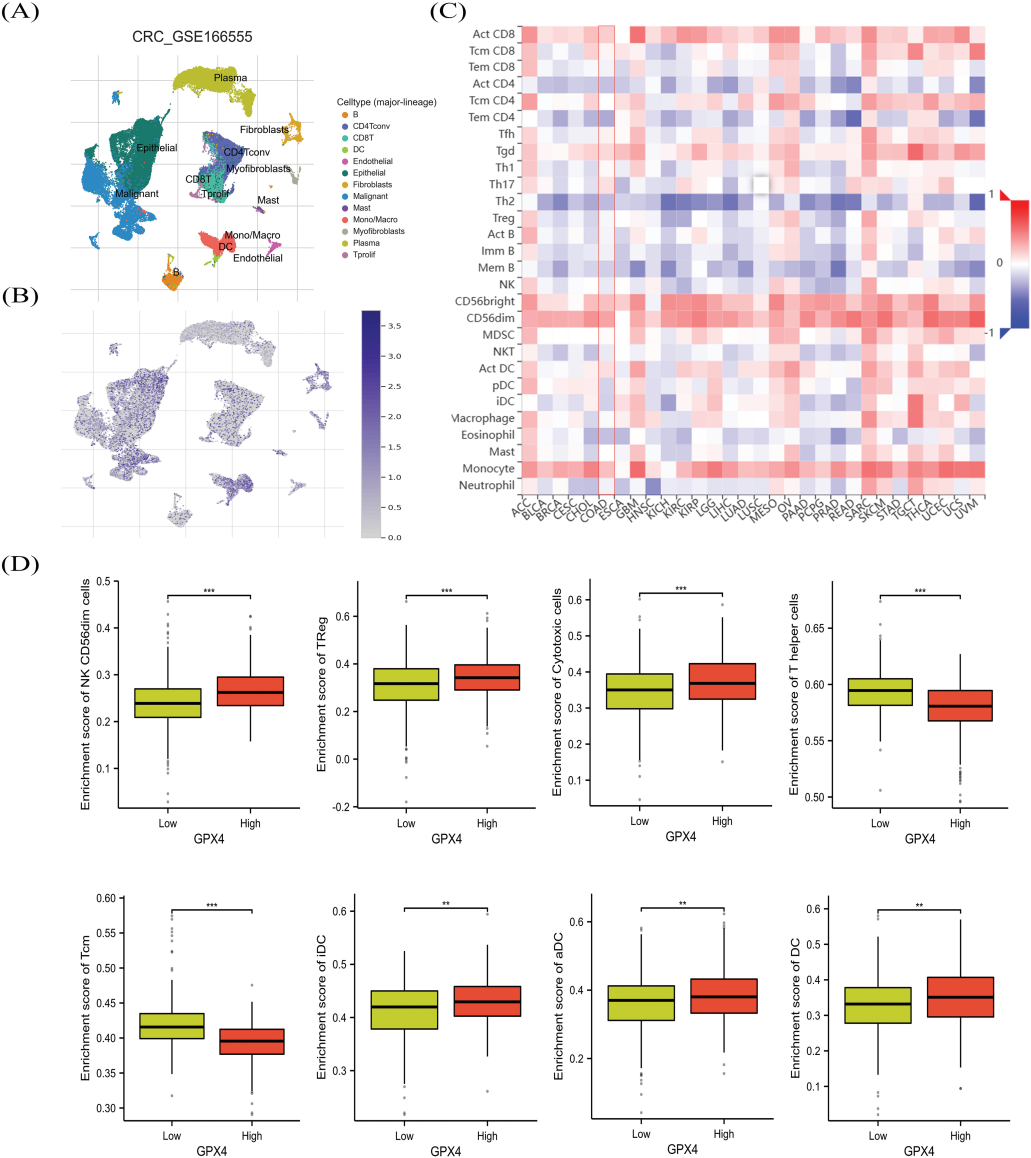
The association between GPX4 and immune cell infiltration in COAD. (**A**,**B**) GPX4 distribution in different cell types was obtained from the GSE166555 dataset. (**C**) Analysis of the relation between GPX4 expression patterns relative to immune cell infiltration by using the TISIDB database. (**D**) Comprehensive evaluation of the relationship between various immune cell types and GPX4 expression in COAD (**p < 0.01; ***p < 0.001).

To evaluate the relationship between GPX4 expression and immune cell infiltration, we first detected the distribution of GPX4 in COAD tissues. We found that GPX4 is abundant in malignant cells, epithelial cells, and monocytes/macrophages in CRC, according to the GSE166555 dataset (Fig. 6A,B). The TISIDB database was applied to confirm the exact types of infiltrating immune cells. As depicted in Fig. 6C, GPX4 was positively correlated with the infiltration of T helper (Th) 17 cells, activated CD8 cells, CD56^bright^ Natural killer (NK) cells, and CD56^dim^ NK cells, as well as Monocytes, whereas it was negatively correlated with effector memory CD4 T cells, activated CD4 T cells, and Th 2 cells. The XIANTAO database showed that GPX4 was positively correlated with Cytotoxic cells, Treg cells, CD56^bright^ NK cells, CD56^dim^ NK cells, Th cells, and Central memory T cells (Fig. 6D; Fig. S3A). As indicated by the TIDE database, elevated GPX4 expression positively correlates with Cytotoxic T-lymphocytes (CTLs) dysfunction, which is associated with poor survival prognosis (Fig. S3B). These results indicate that GPX4 expression correlates with immune cell infiltration in patients with COAD.

## Discussion

As a central regulator of ferroptosis, GPX4 exerts cytoprotective effects through enzymatic reduction of cytotoxic lipid hydroperoxides. Emerging clinical evidence reveals significant associations between GPX4 overexpression and malignant progression across various cancers. In thyroid carcinoma, Chen et al. demonstrated that elevated GPX4 expression correlated with advanced tumor invasion (T3–T4 stage) and metastatic progression (pathologic stages III–IV), establishing its independent prognostic value through multivariate Cox regression analysis [13]. Similar results were also reported by Wu et al., who identified that dual overexpression of SLC7A11 and GPX4 confers remarkable platinum resistance and predicts overall survival in cancer patients [26]. Substantiating these observations, our investigation reveals that consistent GPX4 upregulation in COAD tissues, with its expression pattern demonstrating significant correlation with adverse clinical outcomes.

Intriguingly, recent studies identified that the intracellular distribution of GPX4 manipulates its functions. Mao et al. pointed out that mitochondrial GPX4 and dihydroorotate dehydrogenase are important defense arms for the detoxification of lipid peroxides that are not dependent on GPX4 in the cytoplasm to inhibit ferroptosis [27]. Tadokoro et al. reported that doxorubicin-induced ferroptosis is mediated by mitochondria and showed that doxorubicin accelerated mitochondrial lipid peroxide production, which was effectively rescued by mitochondrial GPX4 [28]. However, it should be emphasized that our study was limited by its exclusive focus on cytosolic GPX4 localization through conventional immunohistochemical analysis. Future investigations employing mitochondrial-cytosolic fractionation coupled with immunoblotting, or confocal microscopy with organelle-specific markers, are warranted to establish the spatial distribution dynamics of GPX4 isoforms.

As a pivotal antioxidant effector, GPX4 is frequently upregulated by cancer cells to counteract oxidative stress and sustain malignant progression. However, the regulatory mechanisms underlying GPX4 regulation in cancer are still unknown. Our bioinformatic analysis identified seven candidate microRNAs (miR-101, miR-214-3p, miR-26a/b, miR-371-3p, miR-495, and miR-657) potentially targeting GPX4 in COAD. Among these candidates, only miR-214-3p has been mechanistically linked to GPX4 suppression in hepatocellular carcinoma via direct 3′UTR binding [29]. This suggests a potential regulatory paradigm, where miRNA-GPX4 interactions may exhibit tissue-specific or tumor-type selectivity, and further studies are required to confirm the interaction between miRNAs and GPX4 in COAD.

An important finding of our study is that GPX4 overexpression likely reduced CTLs infiltration, which could attenuate the response to immunotherapy and strengthen the protection of tumor cells. We also observed that GPX4 expression was negatively correlated with CD4^+^ T cell infiltration. CD4^+^ T cells are the dominant target tumor cells in the immune system, and dysfunction of CD4^+^ T cells can enable adaptive immune resistance, resulting in tumor immune escape and metastasis [30]. A recent study showed that GPX4 inhibitors could enhance the response of cancer patients to immunotherapy by promoting T cell infiltration. For example, Li et al. screened the molecular compound N6F11, which effectively triggered the degradation of GPX4, especially in tumor cells within the tumor microenvironment, but did not induce the degradation of GPX4 in immune cells, to successfully induce protective immunity [31]. This evidence demonstrated that the role of GPX4 is in regulating tumor immune cell infiltration. However, in this particular study, using bioinformatics analysis, we have predicted an inverse correlation between GPX4 expression and cytotoxic T cell abundance, but these computational predictions require further validation. Taken together, our observations, coupled with existing literature, suggest that GPX4-driven poor prognosis may be the result of its dual role in intrinsic tumor cell protection and extrinsic immune suppression.

As expected, we found that knockdown of GPX4 induced cellular senescence in COAD cells. GPX4 as a key driver in the cellular senescence process in COAD cells, reflected by the alteration of the biomarkers p21, Lamin B1, and the secretory phenotype IL-6, while the activity of SA-β-gal. Previous reports indicate that P53 can delay cellular senescence in PC12 cells in an SLC7A11 and GPX4 dependent manner. Knockdown of P53 downregulated SLC7A11 and GPX4 expression, leading to cellular senescence and ferroptosis [15]. Xu et al. showed that the protective effect of 1,25(OH)_2_D_3_ against D-galactose-induced ferroptosis and senescence was reversed by the inhibition of GPX4 (RSL3), demonstrating that GPX4 participates in the cellular senescence process [32]. We hypothesized that GPX4 deficiency causes cells to lack anti-oxidant capacity and makes them more sensitive to oxidative stress, which is considered a common driver of senescence. Nevertheless, further investigations are indispensable to demystify this mechanism.

In summary, our study demonstrated that GPX4 mRNA and protein expression are upregulated in COAD tissues and could predict the prognosis of patients with COAD from our collection of samples. High GPX4 expression was associated with increased infiltration of malignant cells and cytotoxic T-lymphocytes dysfunction. We also performed a series of cell experiments that confirm GPX4 knockdown inhibited the proliferation and induced cellular senescence of CRC cells. This evidence suggests that GPX4 is a potential target gene for COAD treatment and serves as a prognostic biomarker.

## Supplementary Materials



## Data Availability

The datasets presented in this study can be found in online repositories. The names of the repository can be found in the article.

## Abbreviations

COAD: Colorectal adenocarcinoma
GPX4: Glutathione peroxidase 4
CRC: Colorectal cancer
IL-6: Interleukin-6
GAPDH: glyceraldehyde-3-phosphate dehydrogenase
OS: Overall survival
PBS: Phosphate-buffered saline
GBMLGG: Glioma
GEO: Gene Expression Omnibus
GO-KEGG: Gene ontology-Kyoto encyclopedia of genes and genomes
ROC: Receiver operating characteristic curve
TF-miRNA: The protein interaction network and transcription factor-miRNA
CTLs: cytotoxic T-lymphocytes
RT: Room temperature
CCK-8: Cell counting kit-8
RIPA: radio-immunoprecipitation assay lysis buffer
NK: Natural killer
Th: T helper
EdU: 5-ethynyl-2′-deoxyuridine
SA-β-gal: senescence associated **β**-galactosidase
DEGs: Differentially expressed genes
IHC: Immunohistochemistry
SASP: senescence-associated secretory phenotype
BP: Biological Process
CC: Cell Component
MF: Molecular Function
PVDF: Polyvinylidene fluoride
RT-qPCR: Reverse transcription-quantitative polymerase chain reaction

## Supplementary Materials

The supplementary material is available online at https://www.techscience.com/doi/10.32604/or.2025.063395/s1.
