# Supplementary material for: GPX4 predicts poor prognosis and regulates tumor proliferation and senescence in colorectal adenocarcinoma
Source: Oncol Res. 2025 Jul 18;33(8):1933–45. doi: 10.32604/or.2025.063395 (PMC12308247; doi:10.32604/or.2025.063395)
Supplement: Supplementary file 1 [file OncolRes-33-63395-s001.docx]

**Supplementary Figure 1.** Survival correlation and GPX4 expression COAD. (A) The relationship between GPX4 expression levels and OS of various cancers was analyzed by using the Sangerbox database. (B) The relationship between GPX4 expression levels and OS of pan-cancers was analyzed using the Sangerbox website.

**Supplementary Figure 2.** Correlation analysis of differentially expressed genes with GPX4 and quantitative results. (A) Gene expression distribution within the GSE40287 dataset. Circles′ size represents |log_2_ (fold change)| degree. (B) Expression of 10 GPX4-related genes in patients with COAD. (C) GPX4-related genes expression relevance with the expression of GPX4 in COAD. (D) Representative results of 5-ethynyl-2′-deoxyuridine staining. (E) Representative results of immunofluorescence staining (**p* < 0.05; ***p* < 0.01; ****p* < 0.001).

**Supplementary Figure 3.** GPX4 contributes to multiple immune cell infiltration. (A) Relationship between GPX4 expression and various immune cell types in colorectal adenocarcinoma analysis using the XIANTAO platform. (B) OS of patients with cytotoxic T lymphocytes dysfunction and GPX4 protein expression based on GSE39582 and GSE12945 datasets (ns, **p* ≥ 0.05; ****p* < 0.001).
